# Influence of Axial Load on Electromechanical Impedance (EMI) of Embedded Piezoceramic Transducers in Steel Fiber Concrete

**DOI:** 10.3390/s18061782

**Published:** 2018-06-01

**Authors:** Zhijie Wang, Dongdong Chen, Liqiong Zheng, Linsheng Huo, Gangbing Song

**Affiliations:** 1 Department of Underground Engineering, School of Civil Engineering, Southwest Jiaotong University, Chengdu 610031, China; zhjwang@swjtu.edu.cn; 2 Key Laboratory of Coastal and Offshore Engineering, Dalian University of Technology, Dalian 116024, China; chendongdlut@mail.dlut.edu.cn (D.C.); joan@mail.dlut.edu.cn (L.Z.); 3 Smart Materials and Structures Laboratory, Department of Mechanical Engineering, University of Houston, Houston, TX 77204, USA

**Keywords:** steel fiber concrete, axial loads, Structural Health Monitoring (SHM), Electromechanical Impedance (EMI), Lead Zirconate Titanate (PZT), smart aggregates

## Abstract

With the advantages of high tensile, bending, and shear strength, steel fiber concrete structures have been widely used in civil engineering. The health monitoring of concrete structures, including steel fiber concrete structures, receives increasing attention, and the Electromechanical Impedance (EMI)-based method is commonly used. Structures are often subject to changing axial load and ignoring the effect of axial forces may introduce error to Structural Health Monitoring (SHM), including the EMI-based method. However, many of the concrete structure monitoring algorithms do not consider the effects of axial loading. To investigate the influence of axial load on the EMI of a steel fiber concrete structure, concrete specimens with different steel fiber content (0, 30, 60, 90, 120) (kg/m^3^) were casted and the Lead Zirconate Titanate (PZT)-based Smart Aggregate (SA) was used as the EMI sensor. During tests, the step-by-step loading procedure was applied on different steel fiber content specimens, and the electromechanical impedance values were measured. The Normalized root-mean-square deviation Index (NI) was developed to analyze the EMI information and evaluate the test results. The results show that the normalized root-mean-square deviation index increases with the increase of the axial load, which clearly demonstrates the influence of axial load on the EMI values for steel fiber concrete and this influence should be considered during a monitoring or damage detection procedure if the axial load changes. In addition, testing results clearly reveal that the steel fiber content, often at low mass and volume percentage, has no obvious influence on the PZT’s EMI values. Furthermore, experiments to test the repeatability of the proposed method were conducted. The repeating test results show that the EMI-based indices are repeatable and there is a great linearity between the NI and the applied loading.

## 1. Introduction

Distributed short steel fibers in concrete hinder the expansion of microcracks and the formation of macroscopic cracks [1], enhancing the crack resistance of concrete. Steel fiber concrete has been widely used in important projects, such as tunnels, subways, airports, and seismic engineering. The health monitoring of steel fiber concrete structures, like that of other concrete structures, is receiving increasing attention.

Piezoelectric materials have been used in the health monitoring of concrete structures for many years [2,3,4]. The characteristics of fast response, high sensitivity, broad bandwidth, and low cost [5,6,7] enable the detection of small changes in the selected variables (stress, damage) at critical locations [8,9]. Lead Zirconate Titanate (PZT), a type of piezoceramic, is widely used because of its strong piezoelectric effect [10,11]. Annamdas et al. presented an easy method of embedding a PZT sensor in concrete for monitoring either fresh concrete or cured concrete [12]. Soh et al. [13] proposed the formulation of a three-dimensional (3D) interaction model of PZT–structure with the consideration of the mass of both the PZT transducers and the adhesive. A reusable PZT transducer setup for monitoring initial hydration of concrete and structural health was developed by Yang et al. [14]. To overcome the shortcoming of PZT’s brittleness and to offer protection to the PZT core, the concept of “Smart Aggregate” (SA) was proposed for use as an embedded sensor in concrete structures for damage detection, structural health monitoring, and other purposes. The smart aggregates are embedded into the desired locations before the casting of the concrete structure [15]. A smart-aggregate-based approach was proposed by Yan et al. [16] for the structural health monitoring of a concrete shear wall structure. Hou et al. [17] developed an SA-based sensing system, which can monitor the seismic stress of low- and middle-rise building structures under moderate earthquakes. Feng et al. [18] used embedded piezoceramics-based smart aggregates transducers along with the active sensing approach to detect common types of pile damage. A combination of smart aggregates and surface-bonded PZTs was used by Divsholi et al. [19] for structural health monitoring and a one-dimensional Electromechanical Impedance (EMI) model accounting for shear lag between the PZT patch and the host structure was presented by Yang et al. [20].

The sensing technology is a critical part of structural health monitoring [21,22]. EMI enabled by PZT transducers in structural health monitoring receives considerable attention [23,24,25,26,27]. Wu et al. [28] performed an investigation to detect debond in reinforced concrete structures. PZT discs were utilized as sensors and actuators in a pitch–catch mode to generate sensor data. The test results indicated that debond between concrete and rebar and yielding in rebar can be detected. Li et al. [29] proposed the preliminary application of a new type of cement-based piezoelectric sensor developed for monitoring traffic flow. Song et al. [30] developed an over-height vehicle–bridge collision detection and evaluation system using PZT transducers. A simple and versatile measurement system was proposed by Baptista et al. [31] to allow for real-time data acquisition from multiple sensors for structural health monitoring based on the electromechanical impedance technique. Tsangouri et al. [32] used embedded piezoelectric transducers to seal cracks and monitor damage recovery of a concrete healing system. Campeiro et al. [33] carried out impedance-based damage detection under noise and vibration effects.

Until now, there have been many methods to evaluate the service performance and health condition of concrete structures [34,35]. The impedance-based SHM method has been a promising tool for damage identification and is considered a real-time evaluation technique [36]. In 1993, Liang et al. [37] first proposed the theoretical basis for the piezoelectric impedance method used in structural health monitoring, and analyzed the single-degree-of-freedom spring–mass–damping system (SMD) model theoretically. The relationship between the impedance of the piezoelectric element and the impedance of the structure was obtained and demonstrated on a cantilever structure. In 1995, Sun et al. [38] applied the piezoelectric impedance technique to structural damage identification of a fabricated truss. In his experiment, a damage index based on root-mean-square deviation (RMSD) was applied to the piezoelectric impedance technique in the field of structural health diagnosis. Park et al. [39] used the principle of piezoelectric impedance to monitor the bolting of pipelines. High-frequency structural excitation was utilized through surface-bonded piezoelectric sensors/actuators to detect changes in structural point impedance due to the presence of damage. This technique can be applied to the rapid detection of a pipeline system after earthquake and other early damage identification of a pipeline engineering structure. An innovative piezoelectric device named a “smart washer” was proposed by Huo et al. [40] with the impedance method to monitor the pre-stress level of rock bolts and a normalized RMSD index was developed to evaluate the degradation level of the rock bolt pre-stress. Bhalla et al. [41] proposed a new approach for fatigue life assessment of bolted steel joints using the equivalent stiffness determined by surface-bonded piezo-impedance transducers. Previously developed impedance models were revisited by Yun et al. [42] to investigate the effects of sensor and/or bonding defects on the admittance measurement. The feasibility of the modified impedance model for sensor self-diagnosis using the admittance measurements was demonstrated by a series of parametric studies using a simple example of PZT-driven single-degree-of-freedom spring–mass–damper system. Yang et al. [43] presented an electromechanical impedance (EMI) model for health monitoring of cylindrical shell structures to investigate the interaction between the PZT transducers and a typical cylindrical shell structure. 

The influence of axial force on piezoceramic-transducer-based monitoring is crucial for evaluation the health status of structures. The pitch–catch method was employed by Liu et al. [44] to measure the amplitude and velocity of active sensing signals during the loading process. The experimental results demonstrated that the load variation within elastic range still has a significant effect on monitoring signals using an SA-based monitoring system. However, the influence of the axial load on the commonly used EMI-based approach for steel fiber concrete structures has not been studied, to the authors’ best knowledge.

In this paper, the authors proposed to experimentally study the influence of the axial load on EMI-based damage or SHM of steel fiber structures. In addition, the influence of steel fiber content on the EMI values will also be experimentally studied. Standard concrete cube specimens (150 mm × 150 mm × 150 mm) with five different steel fiber contents (0, 30, 60, 90, 120) (kg/m^3^) were casted. For each fiber content, three specimens were fabricated. The strength of the concrete specimens is 50 Mpa. The EM impedance signals were recorded at the axial force values of 0, 20, 40, 60, 80, and 100 kN, which ensured the specimens deformed within the elastic range of strains. The elastic loading range prevents damage to the steel fiber concrete specimens and narrows the influencing factor to only the axial load for a test period when the load is increased from 0 to 100 kN. A smart aggregate with a 15 mm × 15 mm × 1 mm PZT patch as its sensing core was embedded in each specimen for the EMI measurement. During each test period, the PZT EMI measurements were acquired through an impedance analyzer. Testing results clearly demonstrated that the axial load greatly influences the EMI value and the effect of axial load should be considered for EMI-based structural health monitoring of concrete structures. Experimental results also clearly revealed that the fiber content, normally at a small mass and volume percentage, has no obvious effect on the EMI values.

## 2. Smart Aggregate (SA) and the Electromechanical Impedance Method

### 2.1. Smart Aggregate (SA)

**Figure 1:**
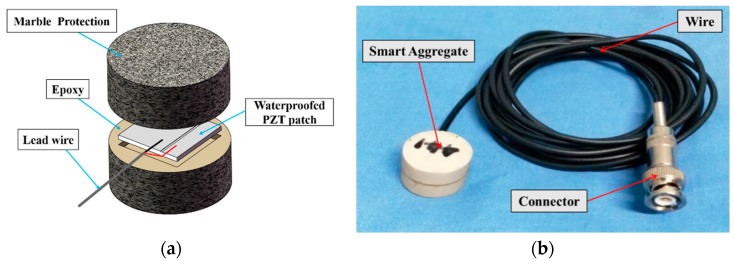
Illustration of Smart Aggregate (SA): (**a**) The composition of the smart aggregate; (**b**) Photo of the smart aggregate.

Shown in Figure 1a is the configuration of a PZT-based smart aggregate (SA). The core of the SA is a waterproofed PZT patch with electrical connecting wires. The dimension of the PZT patch is 15 mm × 15 mm × 1 mm. The PZT patch is sandwiched between two marble blocks, which offer protection to the fragile PZT patch. The height and diameter of the smart aggregates are 20 mm and 25 mm, respectively. Figure 1b shows a photo of the fabricated SA with the connecting wire and a connector. With this configuration, SA can be embedded into a concrete structure and survive the harsh vibration process. The direct and converse piezoelectric effect make the PZT-based smart aggregate suitable for sensing and actuation applications, including active sensing and impedance-based for concrete structure health monitoring. For example, Kong et al. [45] presented research on very-early-age concrete hydration characterization by using piezoceramic-based smart aggregates. Du et al. [46] used SA to monitor quartz Sand-Filled Steel Tube Column (SFSTC) internal stress during impacts.

### 2.2. Electromechanical Impedance (EMI) Method

**Figure 2:**
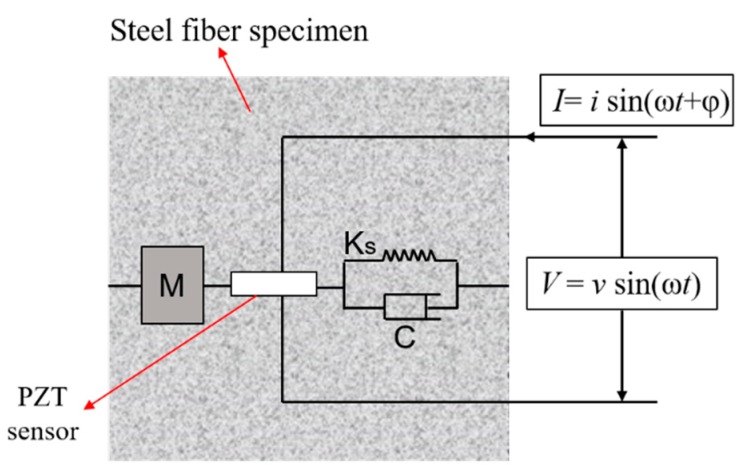
A 1D spring–mass–damper electromechanical model to illustrate the coupling between the PZT and the specimen.

For EMI-based damage detection, a PZT transducer is often embedded in or surface-bonded to a structure. Damage to a structure will often cause the change of its physical properties, such as mass, stiffness, damping, and boundary conditions, and this change will alter the energy transfer between the PZT and host structure [37]. The coupling effect between the host structure and the PZT transducer can be detected by the PZT’s electromechanical impedance (EMI) in the high-frequency range (typically >20 kHz). In this study, the PZT-based Smart Aggregate was embedded into the steel fiber concrete specimen. As shown in Figure 2, a one-degree-of-freedom spring–mass–damper electromechanical system is used to illustrate the coupling effect between the PZT transducer and the steel fiber concrete specimen.

The electromechanical impedance (EMI) and the electric admittance are reciprocal to each other. In the PZT-transducer-driven, single-degree-of-freedom mass–stiffness–damping system, the *Z*(*ω*), which is the impedance of the piezo-transducer coupled to the host structure, is given by

(1) $$Z\left(\omega \right)=\frac{h_{A}}{i\omega w_{A}l_{A}}{\{{\bar{\varepsilon }}_{33}^{\sigma }-\frac{Z_{S}}{Z_{S}+Z_{A}}d_{32}^{2}{\bar{Y}}_{22}{}^{E}\}}^{-1}$$

where ω is the angular frequency of excitation; *i* is the imaginary unit; *w_A_*, *h_A_*, and *l*_A_ are the width, thickness, and length of the PZT patch, respectively; ${\bar{\varepsilon }}_{33}$ is the complex permittivity when the PZT stress is zero or a constant; *d*_32_ is the piezoelectric constant; and ${\bar{Y}}_{22}^{E}$ is the complex Young’s modulus at zero or constant electric field. Please note that the complex Young’s modulus includes a real part that is called the elastic Young’s modulus, and an imaginary part that is called the loss Young’s modulus. *Z_A_* and *Z_S_* represent the electromechanical impedance of PZT and the structure, respectively. Assuming that the mechanical properties of the PZT patch do not vary during the measurement procedure, Equation (1) shows that the electrical impedance of the PZT patch is directly related to the structure’s impedance [47]. Axial force causes changes in the structure’s mechanical impedance, thus changing local dynamic features. Based on the EMI information measured by the impedance analyzer, the root-mean-square deviation of the impedance real part (*RMSD_R_*) is used in this paper, as indicated by

(2) $$RMSD_{R}=\sqrt{\frac{\sum_{N}{\left(Z_{i}^{R}-Z_{N}^{R}\right)}^{2}}{\sum_{N}{\left(Z_{N}^{R}\right)}^{2}}}\times 100\%$$

where $Z_{i}^{R}$ is the *i*th measurement of the EMI real part signal at a certain frequency range, and $Z_{N}^{R}$ is the real part of the EMI signal under the same frequency range in the nondestructive state.

## 3. Steel Fiber Concrete Specimens and Experimental Setup

### 3.1. Steel Fiber Concrete Specimens

**Figure 3:**
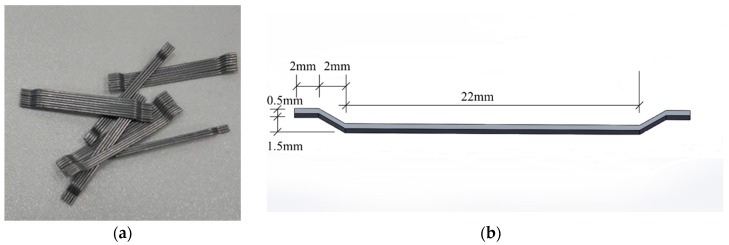
End-hook-type steel fibers used in this research: (**a**) Photo of steel fibers; (**b**) Schematic diagram of steel fiber.

**Figure 4:**
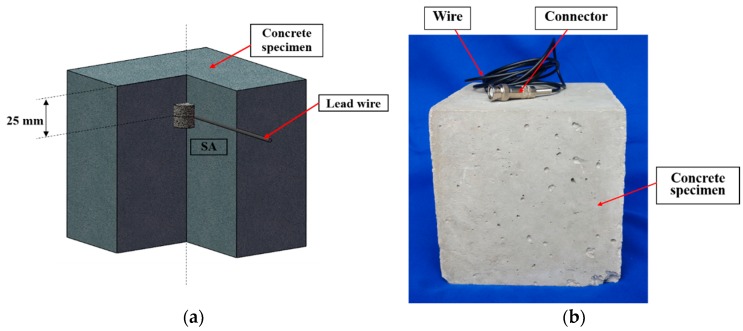
Steel fiber concrete specimen: (**a**) The location schematic diagram of the smart aggregate; (**b**) Photo of concrete specimen.

The cube specimens were composed of 50 MPa strength concrete with different steel fiber content (0, 30, 60, 90, 120) (kg/m^3^). The stress wave propagation velocity in the 50 MPa strength concrete structure is about 4000 m/s. The concrete mix proportion is shown in Table 1. End-hook-type steel fibers, as shown in Figure 3, were used in this research, and the detailed technical information of the steel fibers is shown in Table 2. The content of the concrete specimens and the groups of specimens are shown in Table 3. The dimension of the specimens was 150 mm × 150 mm × 150 mm. The steel fiber concrete specimens were fabricated in the lab in accordance with the fiber concrete specimen mixing standards: “Technology specification for application of fiber-reinforced concrete and steel-fiber-reinforced concrete”. For each specimen, one smart aggregate was embedded in the steel fiber concrete specimen, as shown in Figure 4. The SA is located along the center line of the specimen, 25 mm below the top surface. All test specimens were cured in a standard condition for 28 days and then moved into the lab for testing.

### 3.2. Experimental Setup

**Figure 5:**
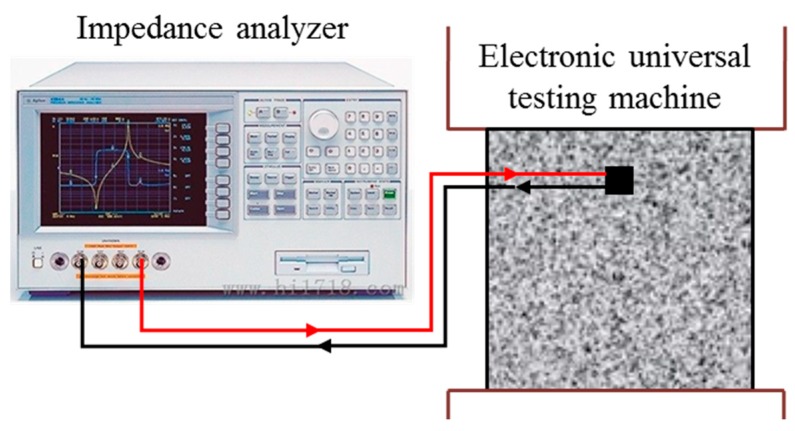
The instrumentation setup.

**Figure 6:**
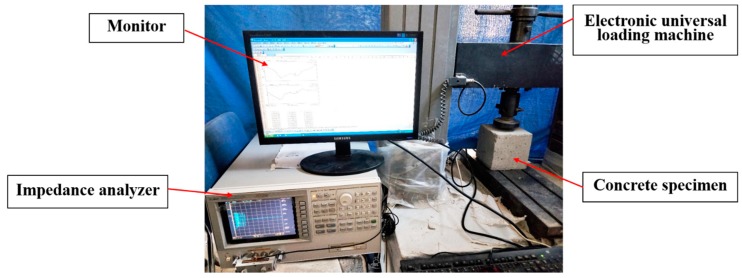
The experimental setup.

The instrumentation setup, mainly including the impedance analyzer, is shown in Figure 5. The entire test setup is shown is Figure 6, which shows that an electric universal loading machine with a capacity of 100 kN was used to load the concrete specimens. A step-by-step load was used in this study to increase the axial load to the specimens. The Agilent 4294A (Agilent Technologies, Santa Clara, CA, USA) precision impedance analyzer produced by Agilent Technologies, was used to measure the impedance signals, the frequency range of which is from 40 Hz to 110 MHz. The excitation frequency in the range of 10 kHz to 500 kHz was chosen in the test. Too low a frequency range is not sensitive to tiny damages in the structures, while too high a frequency range causes PZT to be too sensitive to temperature and boundary conditions, which can affect the judgment of damage information [48].

**Figure 7:**
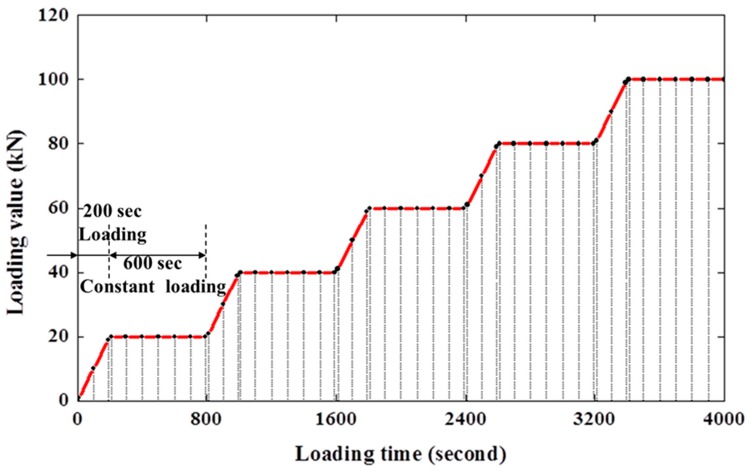
The step-by-step loading schedule.

The baseline impedance signals of all steel fiber concretes were recorded before applying any load. The load was applied on each specimen according to the designed step-by-step load schedule. As shown in Figure 7, the step loads include 0, 20, 40, 60, 80, and 100 kN, and the largest loading value of 100 kN is less than 10% of the failure load of 50 MPa concrete cube specimens; therefore, the strain is within the elastic range. During each load increasing stage, the loading rate was taken as 100 N/s. During this load increasing stage, three EMI measurements were taken at the beginning, the middle, and the end of each stage. Then, the load was kept at a constant for 600 s, during which there are seven measured points and 100 s delay between each measured point. The step-by-step loading strategy is shown in Figure 7.

## 4. Experimental Results and Discussion on Influence of Axial Load on EMI

**Figure 8:**
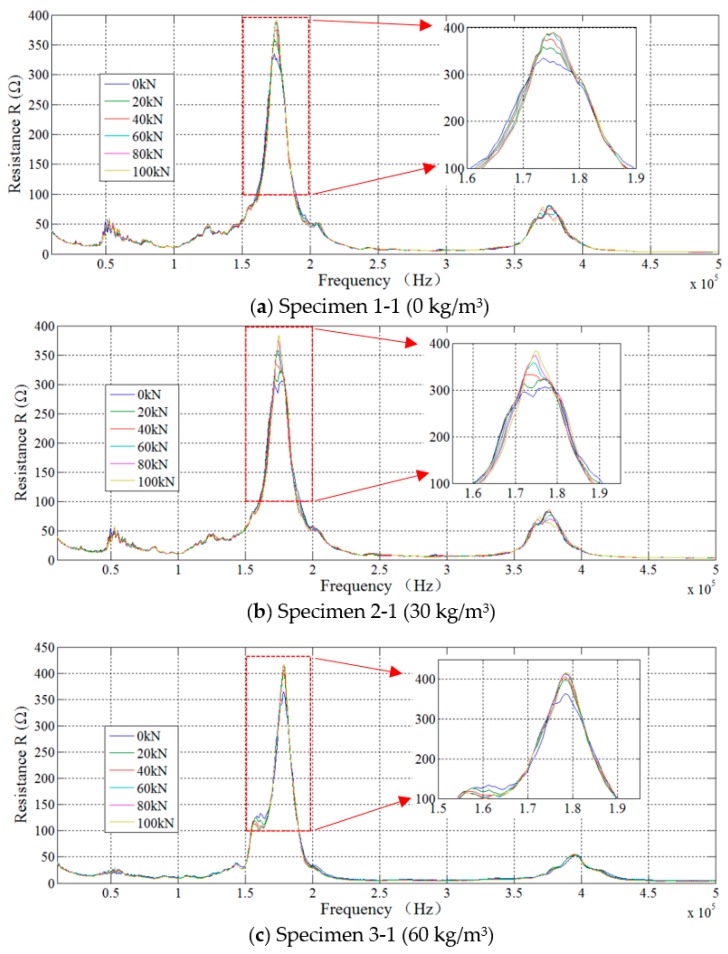

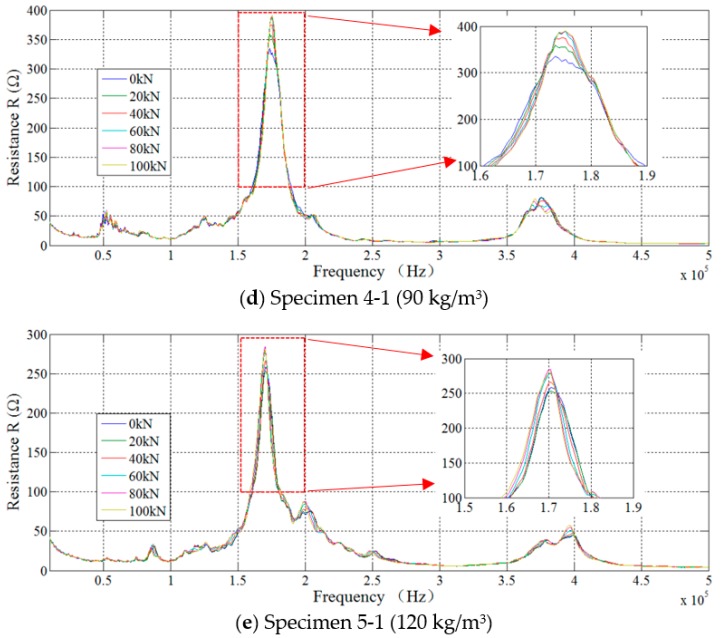
The real parts of the impedance for different specimens.

The step-by-step loading schedule was applied to steel fiber concrete specimens within the elastic range. We purposely chose step-by-step loading so that we could measure the impedance value at a steady state to minimize the dynamic effect. For each loading value, the impedance information of the smart aggregate was directly acquired by the impedance analyzer. The real parts of the impedance for Specimens 1-1, 2-1, 3-1, 4-1, and 5-1 over the frequency range of 10 kHz to 500 kHz were recorded and plotted in Figure 8, which clearly shows that with the increase of the axial load, the EMI value increases. Therefore, the axial load has an influence on the EMI value. Specifically, the central frequencies of Specimens 1-1, 2-1, 3-1, 4-1, and 5-1 are 175 kHz, 174 kHz, 178 kHz, 175 kHz, and 170 kHz, respectively. With the increasing axial forces, the central frequencies shift little. This is caused by the high stiffness and small geometric size. On the other hand, with the increase of the steel fiber content (0, 30, 60, 90, 120) (kg/m^3^), as shown in Figure 8a–e, no clear trend of change of the EMI can be observed. Hence, the steel fiber content has no clear impact on the EMI value, which is reasonable since the steel fiber content in terms of mass or volume percentage is very small. In addition, the steel fibers have very small cross-sectional area.

**Figure 9:**
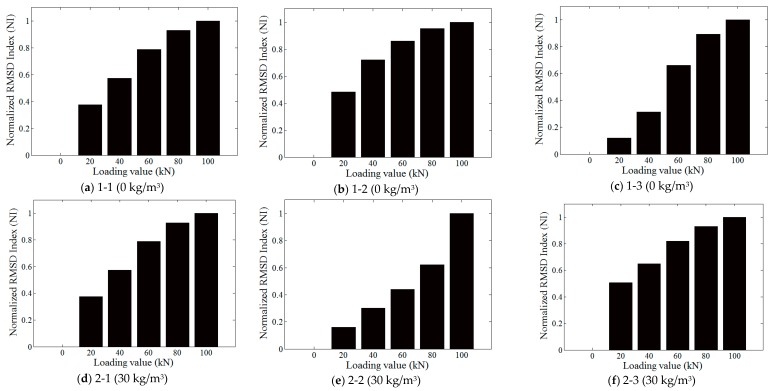

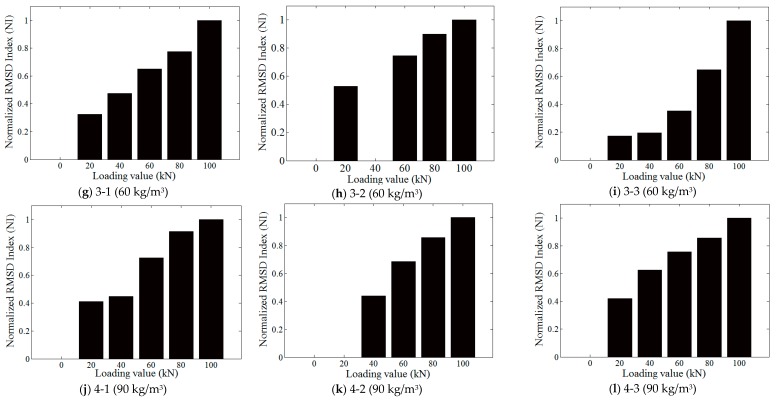

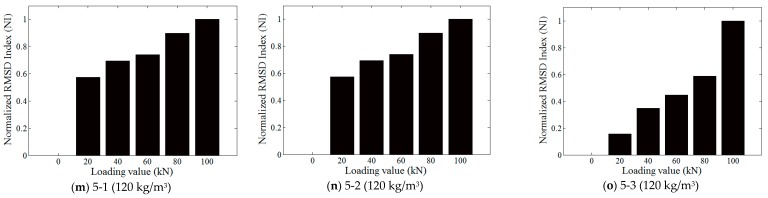
The normalized RMSD index (NI) of all 15 specimens.

The real parts of the recorded impedance values of experiments were analyzed with the RMSD index. Due to the deviation of the RMSD values under different loading conditions, the RMSD results of each specimen were normalized based on the following equation:

(3) $$NI=\frac{I_{RMSD}^{i}-I_{RMSD}^{w}}{I_{RMSD}^{l}-I_{RMSD}^{w}}$$

where *i* denotes the *i*th loading condition in the experiment; $I_{RMSD}^{w}$ is the RMSD index of the specimen without loading applied; $I_{RMSD}^{l}$ is the RMSD index with the largest load applied; and $I_{RMSD}^{i}$ is the RMSD index at the *i*th loading condition. The normalized results are shown in Figure 9.

In all cases in Figure 9, the normalized RMSD indices (NI) increase with the loading value, which is consistent with the finding from Figure 8. It should be noted that the normalized RMSD index in the same mix seems to vary to some degree at the same load level, as shown in Figure 9. There are two main reasons for this: (1) concrete is notorious for its inconsistent properties and uncertainties; and (2) the steel fiber concrete is an anisotropic material. In this research, for each case of fiber content, we have three specimens. It is not surprising for us to see differences among the specimens for each case. The important observation is that all specimens show a clear and similar trend. As an important finding of this research, if an impedance-based index is used for structural health monitoring, the index should be corrected by subtracting the increase caused by the applied load. To address the issue of discrepancy, for practice, we will calculate the mean value of the specimens with the same fiber content, and this mean value will be used for the correction. A future work will be the study of the discrepancy among the specimens with the same fiber content.

In addition, as shown in Figure 9, there is no clear trend in how the fiber content impacts the normalized RMSD index. It is clear that the steel fiber content has no clear impact on the EMI value. In this research, the excitation frequency is in the range of 10 kHz to 500 kHz. The 500 kHz wave has the shortest wave length, 0.008 m or 8 mm, which is obtained by dividing the wave velocity of 4000 m/s by the frequency of 500,000 Hz. We used the fact that the stress wave propagation velocity in the 50 MPa strength concrete structure is about 4000 m/s. Since the profile of the fiber is very small (0.75 mm in diameter) as compared with the shortest wave length of the stress wave (8 mm), the fiber content will have very little impact on the stress wave propagation.

It should be noticed that in Figure 9h,k, the normalized RMSD index (NI) was missing for 40 kN and 20 kN, respectively. The reason for this is that these two impedance signals were acquired during the unstable axial loading stages. Severe drift of impedance signals occurred at 40 kN and 20 kN. Therefore, the two curves cannot be used in calculating the normalized RMSD index (NI).

### Observations and Discussions

Experimental results clearly demonstrated that the axial load greatly influences the EMI value and the effect of axial load should be considered for EMI-based structural health monitoring of concrete structures. Experimental results also clearly revealed that the fiber content, normally at a small mass and volume percentage, has no obvious effect on the EMI values.

To investigate the influence of axial load on concrete, Liu et al. [44] proposed the pitch–catch method to measure the voltage amplitude and the velocity of the monitoring signals during the loading steps. In their experiments, there was no consistent relationship between amplitudes and unloading steps for different specimens or monitoring signals when step-by-step loads were applied on the specimens. However, as shown in Figure 9, the normalized RMSD index (NI)-based impedance method can monitor the loading process well with different steel fiber content (0, 30, 60, 90, 120) (g/m^3^). Therefore, it is more reasonable to use electrical impedance to study the influence of axial force on the voltage amplitude. In this research, the loading is along the axial direction and the loading level is relatively small to ensure the elastic response of the specimen. In reality, the loading may be complex and loading levels may be large and sometimes damage the steel fiber structures. For the next step, we will investigate the feasibility of electromechanical impedance (EMI)-based damage monitoring of steel fiber concrete structures. In addition, we will study the effectiveness of SA-enabled EMI in a complex state of stresses.

## 5. Additional Experiments to Study Repeatability of the Proposed Method

**Figure 10:**
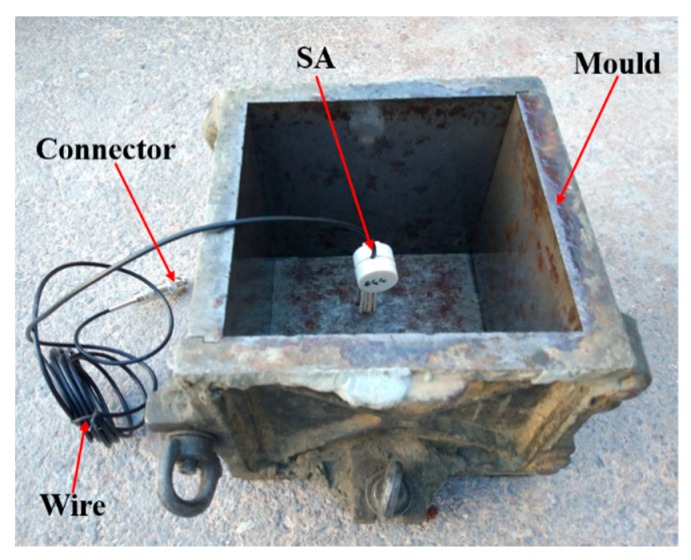
Smart aggregate placement and the mould.

In order to demonstrate the repeatability and reliability of the proposed technique, additional experiments were conducted. A new specimen with 90 kg/m^3^ steel fiber content was casted, as shown in Figure 10. It was cured in a standard condition for 28 days and then moved into the lab for testing.

**Figure 11:**
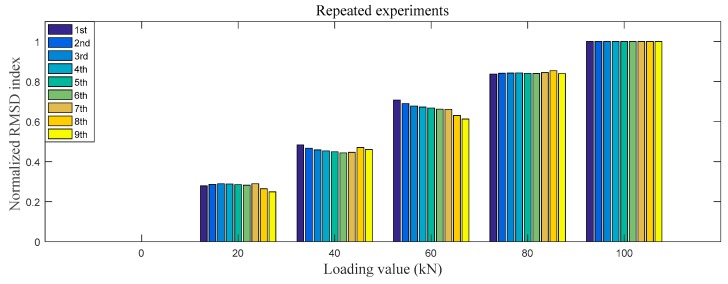
The impedance-based normalized RMSD index of 9 repeated experiments.

The test procedure was the same as that detailed in Section 3.2. Nine repeated experiments were conducted and the impedance-based normalized RMSD index was also calculated according to Equation (2). The impedance-based normalized RMSD index is shown in Figure 11.

As shown in Figure 11, the repeated testing results demonstrate that the normalized RMSD indices vary within a small range for different loading cases. The largest discrepancy is no more than 10%, which is in the loading case of 60 kN. The repeated experimental results clearly show the repeatability of the proposed method.

**Figure 12:**
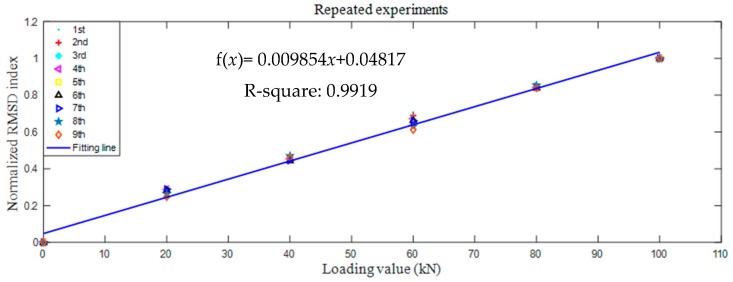
Linear fitting of nine repeated experiments.

In order to demonstrate the reliability of the proposed method, data fitting was conducted for the nine repeated experimental results as shown in Figure 12.

The linear fitting formula f(*x*) = 0.009854*x* + 0.04817 can be obtained. The R-squared value is 0.9919, which means that there is a good linearity between the normalized RMSD index and loading value. These results can be good evidence for the good reliability of the proposed method.

It should be noted that the distribution of steel fibers in steel fiber concrete is uneven [49]. The uneven distribution of steel fiber may cause the RMSD value discrepancy of different specimens with same steel fiber content. In addition, the proprieties of dielectricity and piezoelectricity of each smart aggregate are not the same. Therefore, although with the same steel fiber content, the sensibility of each smart aggregate is different. The above experimental results demonstrated that, for one specific smart aggregate, the stability and good linearity show that the proposed technique has good repeatability and reliability. For field implementation, each smart aggregate will be individually calibrated to obtain the relationship between the EMI value and the applied load.

## 6. Conclusions and Future Work

This paper addressed two important issues related to PZT-enabled electromechanical impedance (EMI)-based structural health monitoring of steel fiber concrete structures. The first is how the axial load affects the EMI values and the second is how the steel fiber content impacts the EMI values. To address these two issues, 50 MPa strength concrete cube specimens (150 mm × 150 mm × 150 mm) with different steel fiber content (0, 30, 60, 90, 120) (kg/m^3^) were fabricated and tested under the axial loads of 0, 20, 40, 60, 80, and 100 kN, which ensured the specimens deformed within the elastic range of strains. The elastic loading range prevents damage to the steel fiber concrete specimens and narrows the influencing factor to only the axial load for a test period when the load is increased from 0 kN to 100 kN. Each specimen was equipped with an embedded smart aggregate with a 15 mm × 15 mm × 1 mm PZT patch as its core. During each test period, the PZT EMI measurements were acquired through an impedance analyzer. Experimental results clearly demonstrated that the axial load greatly influences the EMI value and the effect of axial load should be considered for EMI-based structural health monitoring of concrete structures. Experimental results also clearly revealed that the fiber content, normally at a small mass and volume percentage, has no obvious effect on the EMI values. Experiments to test the repeatability of the proposed method were conducted. The repeating test results show that the EMI-based indices are repeatable and there is a great linearity between the NI and the applied loading. As an important finding of this research, if an impedance-based index is used for structural health monitoring, the index should be corrected by subtracting the increase caused by the applied load. To address the issue of discrepancy in practice, we will calculate the mean value of the specimens with the same fiber content, and this mean value will be used for the correction. Future work will be the study of the discrepancy among specimens with the same fiber content. For the next step, we will investigate the feasibility of electromechanical impedance (EMI)-based steel fiber concrete structure damage monitoring. It will be an interesting topic for future work to study the effect of loading rate or unloading rate on the impedance response. In addition, we will study the effectiveness of SA-enabled EMI in a complex state of stresses.

## Figures and Tables

**Table 1:** Technical properties of steel fiber.

Length (mm)	Diameter (mm)	Aspect Ratio	Tensile Strength (MPa)
35	0.75	46	7.4

**Table 2:** Proportions of concrete mix.

Ingredients of Concrete	Cement	Sand	Stone	Water	Pulverized Fuel Ash	Water Reducing Agent
Weight (kg/m^3^)	400	740	1100	150	50	7.4

**Table 3:** The groups of specimens.

Steel Fiber Content of Concrete (kg/m^3^)	Specimen Numbers
0	1-1, 1-2, 1-3
30	2-1, 2-2, 2-3
60	3-1, 3-2, 3-3
90	4-1, 4-2, 4-3
120	5-1, 5-2, 5-3

## References

[1] Bafghi M.B., Amini F., Nikoo H.S., Sarkardeh H., Bafghi M.B., Amini F., Nikoo H.S., Sarkardeh H. (2017). Effect of Steel Fiber and Different Environments on Flexural Behavior of Reinforced Concrete Beams. Appl. Sci..

[2] Song G., Gu H., Mo Y.L., Hsu T.T.C., Dhonde H. (2007). Concrete structural health monitoring using embedded piezoceramic transducers. Smart Mater. Struct..

[3] Yang J.Y., Chang F.K. (2006). Detection of bolt loosening in C-C composite thermal protection panels: I. Diagnostic principle. Smart Mater. Struct..

[4] Zeng L., Parvasi S.M., Kong Q., Huo L., Lim I., Li M., Song G. (2015). Bond slip detection of concrete-encased composite structure using shear wave based active sensing approach. Smart Mater. Struct..

[5] Song G., Gu H., Li H. Application of the Piezoelectric Materials for Health Monitoring in Civil Engineering: An Overview. Proceedings of the Biennial Conference on Engineering, Construction, and Operations in Challenging Environments.

[6] Dumoulin C., Karaiskos G., Sener J., Deraemaeker A. (2014). Online monitoring of cracking in concrete structures using embedded piezoelectric transducers. Smart Mater. Struct..

[7] Qi B., Kong Q., Qian H., Patil D., Lim I., Li M., Liu D., Song G. (2018). Study of Impact Damage in PVA-ECC Beam under Low-Velocity Impact Loading Using Piezoceramic Transducers and PVDF Thin-Film Transducers. Sensors.

[8] Annamdas V.G.M., Radhika M.A. (2013). Electromechanical Impedance of Piezoelectric Transducers for Monitoring Metallic and Non Metallic Structures: A review of Wired, Wireless and Energy Harvesting Methods. J. Intell. Mater. Syst. Struct..

[9] Lu G., Feng Q., Li Y., Wang H., Song G. (2017). Characterization of Ultrasound Energy Diffusion Due to Small-Size Damage on an Aluminum Plate Using Piezoceramic Transducers. Sensors.

[10] Lam K.H., Lin D.M., Ni Y.Q., Chan H.L. (2009). Lead-free Piezoelectric KNN-based Pin Transducers for Structural Monitoring Applications. Struct. Health Monit..

[11] Dumoulin C., Deraemaeker A. (2017). Real-time fast ultrasonic monitoring of concrete cracking using embedded piezoelectric transducers. Smart Mater. Struct..

[12] Annamdas V.G.M., Yang Y., Soh C.K. (2013). Impedance based Concrete Monitoring using Embedded PZT Sensors. Int. J. Comput. Civ. Struct. Eng..

[13] Madhav A.V.G., Soh C.K. (2007). An electromechanical impedance model of a piezoceramic transducer-structure in the presence of thick adhesive bonding. Smart Mater. Struct..

[14] Yang Y.W., Divsholi B.S., Soh C.K. (2010). A Reusable PZT Transducer for Monitoring Initial Hydration and Structural Health of Concrete. Sensors.

[15] Song G., Gu H., Mo Y.L. (2008). TOPICAL REVIEW: Smart aggregates: Multi-functional sensors for concrete structures—A tutorial and a review. Smart Mater. Struct..

[16] Yan S., Sun W., Song G., Gu H., Huo L.S., Liu B., Zhang Y.G. (2009). Health monitoring of reinforced concrete shear walls using smart aggregates. Smart Mater. Struct..

[17] Hou S., Zhang H.B., Ou J.P. (2012). A PZT-based smart aggregate for compressive seismic stress monitoring. Smart Mater. Struct..

[18] Feng Q., Kong Q., Song G. (2016). Damage detection of concrete piles subject to typical damage types based on stress wave measurement using embedded smart aggregates transducers. Measurement.

[19] Divsholi B.S., Yang Y. (2014). Combined embedded and surface-bonded piezoelectric transducers for monitoring of concrete structures. NDT E Int..

[20] Ong C.W., Lu Y., Yang Y. The Influence of Adhesive Bond on the Electro-Mechanical Admittance Response of a PZT patch Coupled Smart Structure. Proceedings of the Structural Stability and Dynamics—The Second International Conference.

[21] Ou J., Li H. (2010). Structural Health Monitoring in mainland China: Review and Future Trends. Struct. Health Monit..

[22] Song G., Wang C., Wang B., Song G., Wang C., Wang B. (2017). Structural Health Monitoring (SHM) of Civil Structures. Appl. Sci..

[23] Lopes V., Park G., Cudney H.H., Inman D.J. (2000). Impedance-Based Structural Health Monitoring with Artificial Neural Networks. J. Intell. Mater. Syst. Struct..

[24] Mascarenas D.L., Todd M.D., Park G., Farrar C.R. (2007). Development of an impedance-based wireless sensor node for structural health monitoring. Smart Mater. Struct..

[25] Wang D., Wang Q., Wang H., Zhu H. (2016). Experimental Study on Damage Detection in Timber Specimens Based on an Electromechanical Impedance Technique and RMSD-Based Mahalanobis Distance. Sensors.

[26] Wang D.S., Li Z., Zhu H.P. (2016). A new three-dimensional electromechanical impedance model for an embedded dual-PZT transducer. Smart Mater. Struct..

[27] Park S., Yun C.-B., Roh Y., Lee J.-J. (2006). PZT-based active damage detection techniques for steel bridge components. Smart Mater. Struct..

[28] Wu F. (2006). Debond Detection using Embedded Piezoelectric Elements in Reinforced Concrete Structures—Part I: Experiment. Struct. Health Monit..

[29] Li Z.X., Yang X.M., Li Z. (2006). Application of Cement-Based Piezoelectric Sensors for Monitoring Traffic Flows. J. Transp. Eng..

[30] Song G., Olmi C., Gu H. (2007). An overheight vehicle bridge collision monitoring system using piezoelectric transducers. Smart Mater. Struct..

[31] Baptista F.G., Filho J.V., Inman D.J. (2012). Real-time multi-sensors measurement system with temperature effects compensation for impedance-based structural health monitoring. Struct. Health Monit..

[32] Tsangouri E., Karaiskos G., Deraemaeker A., Hemelrijck D.V., Aggelis D. (2015). Crack sealing and damage recovery monitoring of a concrete healing system using embedded piezoelectric transducers. Struct. Health Monit..

[33] Campeiro L.M., Silveira R.Z.D., Baptista F.G. (2017). Impedance-based damage detection under noise and vibration effects. Struct. Health Monit..

[34] Chang P.C., Flatau A., Liu S.C. (2003). Review Paper: Health Monitoring of Civil Infrastructure. Acoust. Speech Signal Process. Newsl. IEEE.

[35] Balageas D., Bourasseau S., Dupont M., Bocherens E., Dewinter-Marty V., Ferdinand P. (2000). Comparison between non destructive evaluation techniques and integrated fiber optic health monitoring systems for composite sandwich structures. J. Intell. Mater. Syst. Struct..

[36] Rabelo D.D.S., Steffen V., Neto R.M.F., Lacerda H.B. (2016). Impedance-based structural health monitoring and statistical method for threshold-level determination applied to 2024-T3 aluminum panels under varying temperature. Struct. Health Monit..

[37] Liang C. (1997). Coupled Electro-Mechanical Analysis of Adaptive Material Systems Determination of the Actuator Power Consumption and System Energy Transfer. J. Intell. Mater. Syst. Struct..

[38] Sun F.P. (1994). Truss Structure Integrity Identification Using PZT Sensor-Actuator. J. Intell. Mater. Syst. Struct..

[39] Park G., Cudney H.H., Inman D.J. (2001). Feasibility of using impedance-based damage assessment for pipeline structures. Earthq. Eng. Struct. Dyn..

[40] Wang B., Huo L., Chen D., Li W., Song G. (2017). Impedance-Based Pre-Stress Monitoring of Rock Bolts Using a Piezoceramic-Based Smart Washer—A Feasibility Study. Sensors.

[41] Bhalla S., Vittal P.A., Veljkovic M. (2010). Piezo-impedance transducers for residual fatigue life assessment of bolted steel joints. Struct. Health Monit..

[42] Yun C.B., Farrar C.R. (2009). Sensor Self-diagnosis Using a Modified Impedance Model for Active Sensing-based Structural Health Monitoring. Struct. Health Monit..

[43] Yang Y., Hu Y. (2007). Electromechanical impedance modeling of PZT transducers for health monitoring of cylindrical shell structures. Smart Mater. Struct..

[44] Liu T., Zou D., Du C., Wang Y. (2016). Influence of axial loads on the health monitoring of concrete structures using embedded piezoelectric transducers. Struct. Health Monit..

[45] Kong Q.Z., Hou S., Ji Q., Mo Y.L., Song G.B. (2013). Very early age concrete hydration characterization monitoring using piezoceramic based smart aggregates. Smart Mater. Struct..

[46] Du G., Zhang J., Zhang J., Song G. (2017). Experimental Study on Stress Monitoring of Sand-Filled Steel Tube during Impact Using Piezoceramic Smart Aggregates. Sensors.

[47] Rabelo D.S., Tsuruta K.M., Oliveira D.D.D., Cavalini A.A., Neto R.M.F., Steffen V. (2017). Fault Detection of a Rotating Shaft by Using the Electromechanical Impedance Method and a Temperature Compensation Approach. J. Nondestrut. Eval..

[48] Sohn H., Farrar C.R., Inman D.J. (2003). Overview of Piezoelectric Impedance-Based Health Monitoring and Path Forward. Shock Vib. Digest.

[49] Gettu R., Gardner D.R., Saldívar H., Barragán B.E. (2005). Study of the distribution and orientation of fibers in SFRC specimens. Mater. Struct..

